# Case report: abemaciclib-induced syndrome of inappropriate antidiuretic hormone (SIADH) without underlying kidney injury in a patient with early-stage estrogen receptor (ER)+ breast cancer

**DOI:** 10.3389/fmed.2023.1338566

**Published:** 2024-01-16

**Authors:** Neil Carleton, Julia Foldi

**Affiliations:** ^1^Women’s Cancer Research Center, UPMC Hillman Cancer Center, Pittsburgh, PA, United States; ^2^Division of Hematology/Oncology, Department of Medicine, University of Pittsburgh School of Medicine, Pittsburgh, PA, United States

**Keywords:** ER+ breast cancer, Abemaciclib, hyponatremia, SIADH, early-stage breast cancer

## Abstract

The CDK4/6 inhibitor, abemaciclib, is now the standard of care adjuvant therapy for patients with estrogen receptor-positive (ER+)/human epidermal growth factor receptor 2-negative (HER2-) tumors at high risk of recurrence. Real-world usage uncovers emerging side effects that may have been previously unreported in clinical trials. Here, we present the clinical course of a patient who developed a syndrome of inappropriate antidiuretic hormone (SIADH) without underlying kidney injury due to abemaciclib use.

## 1 Introduction

The CDK4/6 inhibitor, abemaciclib, in conjunction with endocrine therapy, was approved as adjuvant treatment for patients with ER+/HER2-, node-positive, early breast cancer, at high risk of recurrence in 2021 based on improved invasive disease-free survival in the monarchE trial (1). Common toxicities, as reported in clinical studies and the FDA label, include diarrhea, hematological abnormalities, hepatotoxicity, and interstitial lung disease (2). While laboratory abnormalities occurred, such as increased AST and ALT and hypokalemia, hyponatremia was not reported in the monarchE phase III clinical trial.

Interestingly, abemaciclib is thought to affect renal physiology—effects range from a reversible elevation in serum creatinine without true renal injury to biopsy-proven acute kidney injury (AKI) (3, 4). In the MONARCH3 and monarchE trials, 19 and 11% of patients, respectively, experienced an increase in serum creatinine, which seems to have occurred as an isolated incident without underlying AKI (5, 6). Recent real-world data suggest that increased serum creatinine seen with a small decrease in eGFR occurs in over 60% of patients treated with abemaciclib (7). In some cases, however, biopsy-proven AKI from CDK4/6 inhibitors have been documented, with the majority of cases showing a pattern of acute tubular necrosis on pathologic examination (4). The median time from CDK4/6 inhibitor initiation to the time of AKI was 278 days, which resulted in discontinuation in all cases.

Despite the multitude of side effects, including nephrotoxicity, there have been no reports detailing abemaciclib-induced syndrome of inappropriate antidiuretic hormone (SIADH) or hyponatremia in the absence of underlying AKI; we herein report on this previously undescribed abemaciclib toxicity.

## 2 Case presentation

Consent was obtained from the patient to publish this case report and associated clinical details. Clinical data were collected from the electronic health record.

The patient was diagnosed with invasive ductal carcinoma at the age of 60 years. Her past medical history was significant for hyperlipidemia, chronic obstructive pulmonary disease with a 35 pack-year smoking history, and peripheral vascular disease. She had a family history significant for breast cancer in both her mother and maternal aunt.

She subsequently underwent segmental mastectomy with sentinel lymph node biopsy, which revealed pT1cN1 (1/1 sentinel nodes positive) ER+/PR+/HER2- breast cancer. The tumor had a Nottingham Score of 8/9 with Ki67 of 45%. OncotypeDX recurrence score was 17. The patient completed adjuvant radiation therapy and began adjuvant anastrozole. She was also counseled about starting abemaciclib given the high-risk tumor features (node positive, histologic grade 3, and Ki67 > 20%). Six weeks following anastrozole initiation, the patient started abemaciclib 100 mg twice daily. After 2 months of therapy, the only notable toxicity was grade 1 diarrhea managed with over-the-counter medication.

Three months following abemaciclib initiation, the patient presented to the clinic with asymptomatic hyponatremia (129 mmol/L; down from a baseline of 141 mmol/L) in the absence of kidney injury (serum creatinine 0.9 mg/dL; baseline 0.9 mg/dL) or other electrolyte abnormalities (K+ = 4.4 mmol/L; baseline 4.2 mmol/L). On examination, she was euvolemic. Further workup showed serum hypoosmolality (258 mOsm/kg), urine osmolality of 370 mOsm/kg, and urine Na + of 68 mmol/L with persistently normal serum potassium, TSH, cortisol, blood urea nitrogen, and no acid/base disturbance. After uninterrupted hyponatremia with a nadir of 125 mmol/L, she was referred for nephrology consultation and was diagnosed with SIADH. Given that SIADH can be associated with advanced malignancy, complete staging scans including CT of the chest, abdomen, and pelvis, a bone scan, and an MRI of the brain were obtained and were all without evidence of breast cancer recurrence or other malignant process. It was felt that drug-induced SIADH related to abemaciclib use was the most likely etiology, ruling out the patient’s other medications (trazodone, tylenol, and anastrozole) as potential causes.

**Figure 1:**
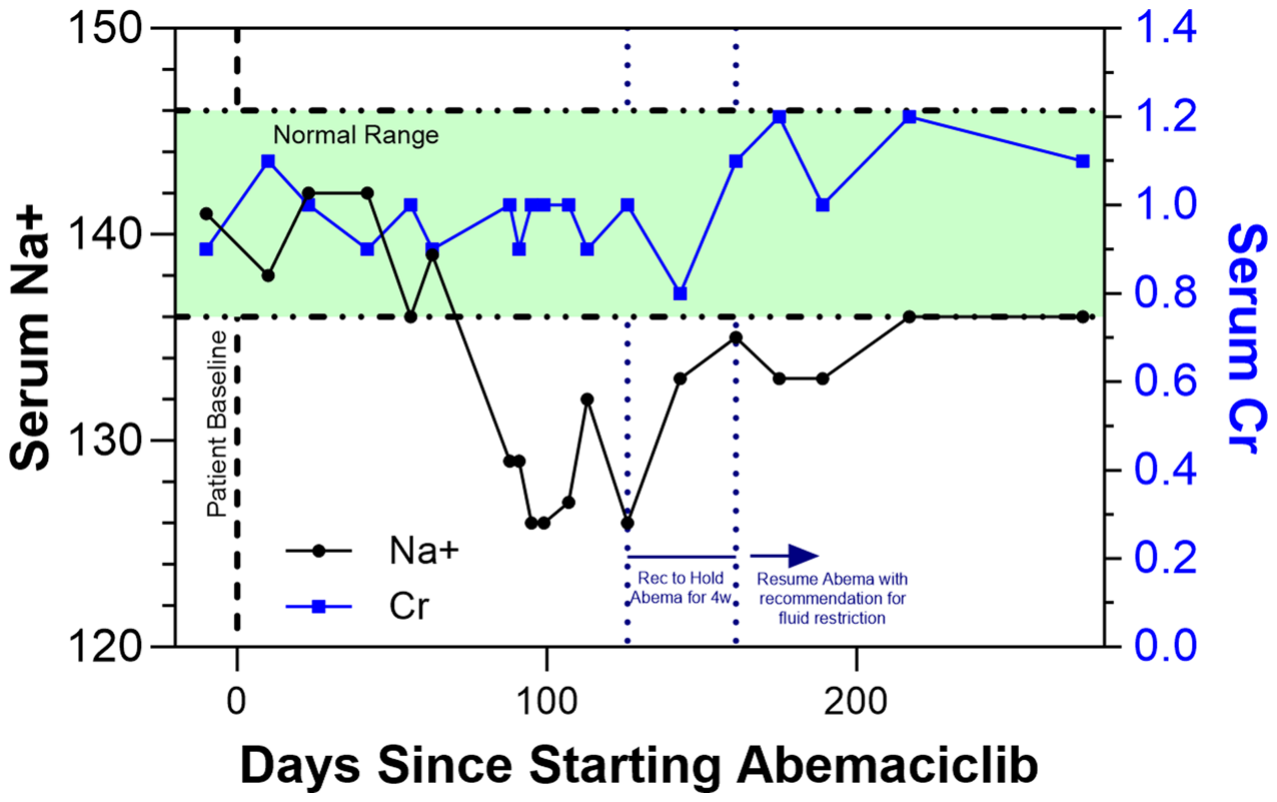
Shows the trends in serum Na+ (black line) and serum creatinine (Cr; blue line) as a function of days since the patient started adjuvant abemaciclib. After the onset of SIADH and hyponatremia, it was recommended to hold abemaciclib for 4 weeks, which resulted in a normalization of serum Na+ levels. The patient has since resumed and continues to take abemaciclib with a recommendation for fluid restriction.

Management included a recommendation to hold abemaciclib for 4 weeks, which resulted in a near-resolution of hyponatremia (Figure 1) as well as fluid restriction (8). As she remained asymptomatic, abemaciclib was restarted at the same dose per patient preference, along with fluid restriction with a suggested starting goal of less than 1 L per day. Commencement again resulted in slightly worsening hyponatremia, further supporting abemaciclib-induced toxicity.

As the patient adjusted to fluid restriction, the hyponatremia again appeared to resolve without abemaciclib discontinuation or symptomatic issues. Ongoing guidance for the patient continues to include fluid restriction; she continues on abemaciclib with close clinical monitoring and remains disease-free with clinical surveillance per appropriate guidelines. Nearly 300 days following abemaciclib initiation, the patient’s renal function has remained stable and the sodium disturbance seems to have fully resolved.

## 3 Discussion

We describe a case of abemaciclib-induced SIADH occurring in the absence of kidney injury. Based on our literature review, there are no other reported cases of this phenomenon. This finding is significant given the increasingly widespread use of abemaciclib in both the adjuvant and metastatic setting (9). While hyponatremia was not reported in the monarchE study, prior studies such as MONARCH1 reported hyponatremia in up to 20% of patients (unknown whether or not these cases occurred in the absence of AKI) (10). Interestingly, the hyponatremia in the patient described here developed after months of treatment, which seems aligned with prior reports that show patients who develop AKI do so in a delayed fashion as well (4).

SIADH is known to be a disorder of impaired water excretion. In most cases, elevated ADH leads to water retention and the development of hyponatremia. In this case, the patient developed hyponatremia nearly 100 days following abemaciclib initiation workup to confirm the SIADH diagnosis included serum and urine electrolytes as well as serum and urine osmolality. Repeated laboratory values included normal TSH and cortisol, serum hypoosmolality, and a urine osmolality above 100 mOsm/kg, which are all consistent with SIADH.

Given the delay in SIADH onset, we sought further workup for etiology given that drug-induced SIADH is a diagnosis of exclusion. SIADH can occur in the setting of advanced malignancy through ectopic ADH secretion, most commonly in patients with small-cell lung cancer. It is uncommon for primary or metastatic breast tumors to ectopically secrete ADH, and the patient did not present with SIADH at the time of diagnosis but following adjuvant therapy initiation. However, to rule out a breast cancer recurrence or a second primary malignancy, we pursued imaging with CT of the chest, abdomen, and pelvis along with MRI of the brain, which were all negative.

All of the patient’s medications were reviewed to determine whether any of those may be contributing to SIADH development. Concurrent medications included trazodone and a proton pump inhibitor; both have been weakly associated with the development of drug-induced SIADH. However, given the fact that the patient had been chronically on the medications for many years prior to the development of SIADH, it is unlikely that these medications were etiologically relevant. Her only other new medication was anastrozole, which has never been described to be associated with hyponatremia or SIADH. Given the delayed time course, which is consistent with prior reports on CDK4/6 inhibitor-mediated kidney injury, and the restoration of normal serum sodium levels after holding abemaciclib for 4 weeks, we determined that abemaciclib-induced SIADH was most likely.

The pathophysiology of drug-induced SIADH is thought to be caused by nephrogenic antidiuresis in the setting of increased aquaporin-2 (AQP2) upregulation in the collecting duct cells. In this case, upregulation enhances osmotic water reabsorption. This is believed to be the most common cause of hyponatremia in the setting of anticancer chemotherapeutic agents (11); whether this also occurs in the setting of abemaciclib-induced SIADH is to be determined.

Fluid restriction and medication discontinuation are the mainstay of treatment for patients with drug-induced SIADH. The patient was counseled on dose-reducing or stopping the abemaciclib altogether. Given that she was asymptomatic, she was not in favor of either approach and preferred to continue on at the same dose (100 mg) of abemaciclib. We then recommended, in consultation with nephrology, fluid restriction with a goal of less than 1 L per day. Following this approach, the patient’s serum sodium has normalized even while continuing abemaciclib.

In conclusion, we present a case of abemaciclib-induced SIADH in the absence of kidney injury in a patient with node-positive, ER+ breast cancer. While both elevated serum creatinine and acute kidney injury have been reported in the setting of abemaciclib use, to our knowledge, this is the first report of abemaciclib-induced SIADH with hyponatremia in the absence of elevated serum creatinine and kidney injury.

## Data availability statement

The original contributions presented in the study are included in the article/supplementary material, further inquiries can be directed to the corresponding author.

## Ethics statement

Written informed consent was obtained from the individual(s) for the publication of any potentially identifiable images or data included in this article.

## Author contributions

NC: Data curation, Formal analysis, Funding acquisition, Investigation, Visualization, Writing – original draft, Writing – review & editing. JF: Conceptualization, Data curation, Formal analysis, Investigation, Methodology, Project administration, Resources, Supervision, Visualization, Writing – review & editing.
